# Anti-Asian sentiment on social media and mental health among Asian and Asian American populations in the United States during the COVID-19 pandemic: A systematic literature review

**DOI:** 10.1016/j.ssaho.2025.102282

**Published:** 2025-11-28

**Authors:** Chulwoo Park, Chelsea Gamboa Lingao

**Affiliations:** a Department of Sociology, Anthropology, and Public Health, University of Maryland Baltimore County, Baltimore, MD, 21250, United States; b Department of Public Health and Recreation, San José State University, San Jose, CA, 95192, United States

**Keywords:** Anti-Asian sentiment, Xenophobia, Social media, Discrimination and racism, Mental health, COVID-19

## Abstract

Asian and Asian American populations in the United States represent the fastest-growing group, with an 81% increase between 2000 and 2019. During the COVID-19 pandemic, they were targeted by anti-Asian racism, leading to experiences of discrimination, stigmatization, verbal harassment, and physical attacks. The purpose of this study was to conduct a systematic literature review to understand how anti-Asian sentiment, particularly through social media, affected the mental health of Asian populations. We established six key areas as eligibility criteria: (1) Asian populations, (2) the COVID-19 pandemic, (3) racism and discrimination, (4) mental health, (5) the role of social media, and (6) the United States. Using five online databases—PubMed, Web of Science, CINAHL, ScienceDirect, and OpenGrey—we applied the PRISMA 2020 flow diagram, which resulted in 15 articles for review. A total of four themes emerged. First, during the COVID-19 pandemic, Asian and Asian American populations experienced high rates of racial prejudice and discrimination, which were widely discussed on social media. Second, there was no geographic pattern distinguishing rural and urban areas in terms of anti-Asian hate incidents. Third, prejudice and discrimination had a negative impact on mental health. Lastly, social media served as both a platform for spreading and combating anti-Asian hate crimes. Future studies should examine how social media particularly influences offline hate crimes and explore culturally tailored interventions to reduce racism and discrimination among Asian and Asian American populations, ultimately promoting community resilience.

## 1. Introduction

### 1.1. Rationale

The Asian and Asian American populations in the United States, the fastest-growing racial or ethnic group, expanded by 81% from 10.5 million in 2000 to 18.9 million in 2019, reflecting remarkable growth and diversity (Budiman & Ruiz, 2021). Comprising of a variety of communities, including Chinese, Indian, Filipino, Vietnamese, Korean, and Japanese, these groups collectively make up 85% of the Asian American populations, showcasing their distinct cultural and social characteristics (Budiman & Ruiz, 2021). Despite the Asian American community growing at such a high rate, historically, mental health needs among these individuals are understudied and underfunded (Bui & Lau, 2024). Limited opportunity to investigate issues and experiences that arise can be attributed to anti-Asian biases, for example, in federal grant review processes with less than 1% of the National Institutes of Health’s funding portfolio supporting scientific investigations involving this community (Yip et al., 2024).

Mental health continues to be a significant aspect of well-being that has attracted an increasing amount of attention, particularly as the literature investigates the extent to which it affects quality of life and how certain communities are subjected to an undue burden. Mental health outcomes, including substance use, anxiety, and depression, were particularly prevalent at the onset of the COVID-19 pandemic (Cheslack-Postava et al., 2024). Although Asians and Asian Americans initially reported a lower prevalence of mental health symptoms (Yeh & Tsai, 2024), they reported higher levels of psychological distress during and after the COVID-19 pandemic (McGarity-Palmer et al., 2023). Suicide rates among Asian American and Pacific Islander youth are alarmingly high, with suicide being the leading cause of death for this demographic. This can be attributed to a variety of factors, such as racialized stress, xenophobic sentiment, and personal ideas aligning with the “model minority” (Bui & Lau, 2024; Yeh & Tsai, 2024). While Asian and Asian American individuals generally report lower prevalence rates of mental health symptoms and utilization of mental health services than other racial groups, the research gap, underfunding, and anti-Asian biases, as aforementioned, have been documented (Bui & Lau, 2024; Yeh & Tsai, 2024; Yip et al., 2024). The overall exacerbated mental health needs across various communities during the COVID-19 pandemic can be attributed to the rise in racism and the subsequent increase in the prevalence of race-related stress. Nevertheless, it has not yet been determined whether this association is sustained by racism and anti-Asian sentiment that are exclusively observed and experienced on social media through a systematic literature review approach.

The COVID-19 pandemic has recently shed light on the limited exploration of anti-Asian racism in the United States. As Kim and Sundstrom (2023) described, this can be attributed to the Black-White binary of American racial politics. By categorizing racism into merely two categories, it ultimately harms all racial minorities, refusing to acknowledge the nature of diversity and equity, including individuals of Asian descent who do not fall under either of those categories. This is also reinforced when discussing Asians and Asian American demographics, where Asians, despite the many ethnicities that would fall under this category, are minimized to the single category of “Asian,” thereby simplifying cultural and ethnic identities to fit on a spectrum of a racial hierarchy, as (Kim, 1999) argued. This neglect marginalizes the unique challenges faced by Asian Americans, with xenophobia frequently labeling people of Asian descent as “perpetual outsiders,” leading to social and cultural exclusion. It can also obstruct the provision of adequate and appropriate support for Asians and Asian Americans by reducing all Asian individuals, who can identify with one or more of several hundred Asian ethnicities, to a single identity. Furthermore, discrimination against Asian Americans is multifaceted, encompassing both overt racism and more complex dynamics, such as distorted positive perceptions or xenophobia.

The roots of this discrimination date back to the 1850s, when Chinese immigrants were stereotyped as “perpetual foreigners.” This perception led to the Chinese Exclusion Act of 1882, the first law to restrict immigration to the United States based on ethnicity (National Archives, 2021a). Similarly, during World War II, over 122,000 Japanese American men, women, and children were forcibly relocated to internment camps, despite a lack of evidence proving them to be a threat (National Archives, 2021b). Historical examples, such as the Chinese Exclusion Act and Japanese American internment during World War II, illustrate the deeply entrenched nature of such discrimination, underscoring the critical need to address anti-Asian racism as a distinct issue (Kim & Sundstrom, 2023). Many endured these injustices in silence and sought justice only decades later (Muramatsu & Chin, 2022). These historical injustices laid the groundwork for contemporary anti-Asian hate crimes and discrimination, which encompass a range of actions and attitudes that continue to marginalize the community, often rooted in historical prejudice and cultural biases. Such behaviors, including verbal harassment, physical attacks, and exclusion, are fueled by stereotypes and the perception of Asian Americans as perpetual foreigners. Kim and Sundstrom (2023) highlighted that the “model minority” myth portrays Asian Americans as universally successful, oversimplifying their experiences and obscuring the real challenges many face. This stereotype not only disregards the diversity within Asian American communities but also minimizes the systemic barriers they encounter. By framing success as the result of purely individual effort, it diverts attention from broader issues of racism and inequality that shape their lives, which can also manifest in a critical gap in research and resources.

Building on the historical context, the COVID-19 pandemic amplified anti-Asian discrimination, fueled by rhetoric linking the virus’s origin to China. Many Asian Americans faced stigmatization, verbal harassment, and an alarming increase in hate crimes. Terms like the “Chinese Virus” from influential figures such as President Trump, contributed to a rise in xenophobia and physical assaults against Asian Americans (Lantz & Wenger, 2023). This harmful language unfairly blamed Asians for the pandemic, fostering anxiety, fear, and a pervasive sense of insecurity. Anti-Asian sentiment, as defined by He et al. (2022), is “antagonistic speech that is directed towards an Asian entity (individual person, organization, or country), and others the Asian outgroup through intentional opposition or hostility in the context of COVID-19.” Anti-Asian sentiment was characterized in this review most accurately by this definition. Such rhetoric not only intensified racial discrimination but also perpetuated global biases against Asian individuals, reinforcing harmful stereotypes and exclusionary practices (Cho et al., 2020; Tsai et al., 2020). Discrimination against Asian Americans extended into workplaces where frontline workers faced microaggressions and stereotyping that heightened their invisibility and expendability. This treatment led to job strain, reduced satisfaction, and adverse physical and mental health outcomes (Le, 2021). The impact of these terms was starkly evident in the surge of hate crimes against Asians. According to Yang et al. (2024), hate crimes against Asian Americans increased by 149% in 2020 and experienced a further surge of 339% from 2020 to 2021. These incidents highlighted the pandemic’s role in magnifying pre-existing racial disparities and creating new avenues for discrimination. Reports also indicated that social media platforms and biased public health messaging played significant roles in spreading anti-Asian sentiment, normalizing discrimination in both online and offline spaces (Cho et al., 2020; Shimkhada & Ponce, 2022). This rise in hate and prejudice underscored the urgent need for culturally sensitive public health campaigns and community support initiatives. Scholars have argued that addressing the root causes of such discrimination requires a multi-pronged approach, including policy changes, education, and fostering cross-cultural understanding to mitigate the public health toll of racialized rhetoric (Gao & Liu, 2021; Viladrich, 2021).

Race-related stress factors and experiences have been associated with negative health outcomes. Race-based stress refers to psychological distress developed from exposures to negative race-based incidents (J. P. Yang et al., 2024). In the additional context of COVID-19, this has been associated with increased psychological distress, depression and anxiety (McGarity-Palmer et al., 2023). Given that negative race-based experiences increased during the pandemic, manifesting in the form of anti-Asian hate crimes, or anti-Asian rhetoric and sentiment online and among communities (Chen et al., 2022; Cho et al., 2020; Jacques et al., 2023; Ng & Indran, 2024; Nguyen et al., 2020; Pan et al., 2021; Wei et al., 2024), it is critical to further examine the relationship of racism experienced online and mental health symptoms. Some studies have particularly investigated the role of online activity on health and well-being. Social media has been observed to be a common coping tool, for example, in response to discrimination, or as a form of seeking social support (Wheeler et al., 2023; C. Yang et al., 2020). It has also been observed to be a vessel for vicarious discrimination, which significantly predicted the prevalence of race-based stress symptoms (Wheeler et al., 2023) and was found to be associated with elevated levels of depression and anxiety (Chae et al., 2021).

This recent surge in anti-Asian rhetoric and sentiment, even as the pandemic has been declared “over,” calls for careful reflection to better understand its broader implications and to inform efforts that can reduce the likelihood of similar harms affecting other communities in the future. Summarizing and examining the existing literature through the lens of its impact on Asian Americans’ mental health offers a foundation for understanding the consequences of discrimination and for guiding future research and responses.

### 1.2. Objectives

Although significant work has explored social media-driven anti-Asian sentiment during the pandemic, a critical gap remains in understanding how these online interactions translate into measurable mental health outcomes for Asian and Asian American communities. Specifically, the link between exposure to discriminatory content and mental health conditions, such as anxiety and depression, remains insufficiently examined. This gap raises the following important question: How did social media-driven anti-Asian sentiment during the COVID-19 pandemic affect the mental well-being of Asian and Asian American populations? Addressing this question provides an opportunity to explore the tangible impacts of discrimination and uncover actionable insights to support these communities. In this context, our research uniquely addressed racism, which will be used interchangeably with anti-Asian sentiment against Asian populations, particularly during COVID-19, by synthesizing findings from studies analyzing social media conversations and public attitudes. This synthesis provided a comprehensive summary of how anti-Asian sentiment and discrimination on social media impact the mental health of Asians and Asian Americans and public health, revealing patterns and gaps that might otherwise remain obscured.

## 2. Methods

### 2.1. Eligibility criteria

The inclusion criteria for this review required studies to address six key areas: 1) Asian populations, 2) the COVID-19 pandemic, 3) racism and discrimination, 4) mental health impacts, 5) the role of social media as a platform, and 6) contextual relevance to the United States. The racism and discrimination criteria may also be fulfilled by eligible articles if they were to include discussion of anti-Asian sentiment, which is critical to our research objective. Articles were not required to involve incidents of a criminally liable nature, but needed to include anti-Asian sentiment in order to review the mental health implications of it under the context of the COVID-19 pandemic. Eligible studies were required to be published in English, with the timeframe extending up to October 8, 2024, when the search strategy was established. Articles with any study designs were considered for inclusion, provided they presented original research or relevant secondary analyses aligning with these areas. Only peer-reviewed or editor-reviewed articles published in academic journals were finally included to ensure a broad range of high-quality evidence, such as professional expertise, data, community perspectives and robust methodologies. Studies were excluded if they did not directly address Asian populations, lacked focus on the COVID-19 pandemic, or failed to explore the intersection of racism, discrimination, mental health, and social media. Mental health, as discussed in this study, refers to symptoms of depression, anxiety, and psychological distress. Additionally, studies conducted outside the United States or without clear relevance to the United States context were excluded. For synthesis, the included studies were analyzed across six areas to provide a comprehensive understanding of the research focus. These areas included the experiences of racism and discrimination among Asian populations during the COVID-19 pandemic, the mental health impacts of these experiences—such as psychological distress and coping mechanisms—and the role of social media as a platform for solidarity, support, or the spread of misinformation. Additionally, studies exploring the intersection of racism, mental health, and social media were reviewed to understand their interconnected effects. Finally, research with contextual relevance to the United States was examined to tie findings to broader societal patterns and policy implications. This thematic analysis allowed for the identification of trends, gaps, and insights within the body of evidence.

### 2.2. Information sources and search strategy

The search was conducted across five online databases: PubMed, Web of Science, CINAHL, ScienceDirect, and OpenGrey. This multi-database approach ensured the capture of relevant and diverse studies that addressed the research objectives comprehensively. The search strategy was tailored to ensure the inclusion criteria of studies that explored the experiences of Asian populations during the COVID-19 pandemic. It specifically focused on issues of racism and discrimination, their mental health impacts, and the role of social media while maintaining relevance to the United States. This comprehensive and targeted approach allowed the search to yield a broad yet relevant set of records for further screening and analysis. The search strategy was meticulously designed to ensure comprehensive alignment with six key groups listed in the inclusion criteria above. To achieve this, combinations of keywords were used with search field tabs or subject headings, such as Medical Subject Headings (MeSH) and Text Words (tw) for PubMed, as well as MH (searches both major and minor headings) and MM (searches just for major headings) for CINAHL, along with Boolean operators (AND and OR), to identify studies addressing the intersection of these areas. Keywords such as “Asian,” “COVID-19,” “racism,” “discrimination,” “mental health,” “social media,” and “United States” were carefully combined to maximize the precision and relevance of the search results. The detailed search strategies for each online database are introduced in Appendix A.

### 2.3. Selection process

The process of identifying, screening, and selecting articles followed a structured and rigorous methodology to ensure the reliability and validity of the final set of studies included in the review. Two reviewers (CL and an anonymous research assistant) collaborated throughout the entire selection process, which included identifying articles from each database, screening titles, abstracts, and full-texts, and finalizing the inclusion of articles. They jointly designed and applied the search terms, screened the identified records for relevance to the research objectives, and evaluated the articles based on predefined inclusion criteria. This collaborative effort minimized bias and ensured consistency across each stage of the selection process. During the title and abstract screening phase, articles were thoroughly reviewed to determine their alignment with the six groups. If the article title did not appear relevant to our study, the article was excluded. During the following stages, screening by abstract, followed by screening by full text, reviewers screened and excluded titles at the respective stages based on whether they met the search criteria. If there was a disagreement among the two reviewers in whether an article met the requirements sufficiently, then they may be selected to be reviewed for full-text review, upon consultation with the primary investigator to check if all criteria were met. If they were found to still not meet at this latter stage, they were excluded for analysis. Records that were not directly relevant were excluded, while those deemed potentially eligible were further evaluated through full-text reviews. If articles at the screening by abstract phase did not have an abstract available, they automatically moved to the screening by full-text phase. The study investigator (CP) provided oversight by reviewing and validating the search strategy, monitoring each stage to ensure adherence to the research protocol and methodological rigor, and resolving disagreements between the two reviewers. By closely supervising the work of the two reviewers, the study investigator ensured that all included studies met the eligibility criteria and aligned with the research objectives. Ultimately, 15 studies were included in the final review, providing diverse and meaningful perspectives on the experiences of Asian populations during the COVID-19 pandemic in an online context.

## 3. Result

### 3.1. Study selection

**Fig. 1.**
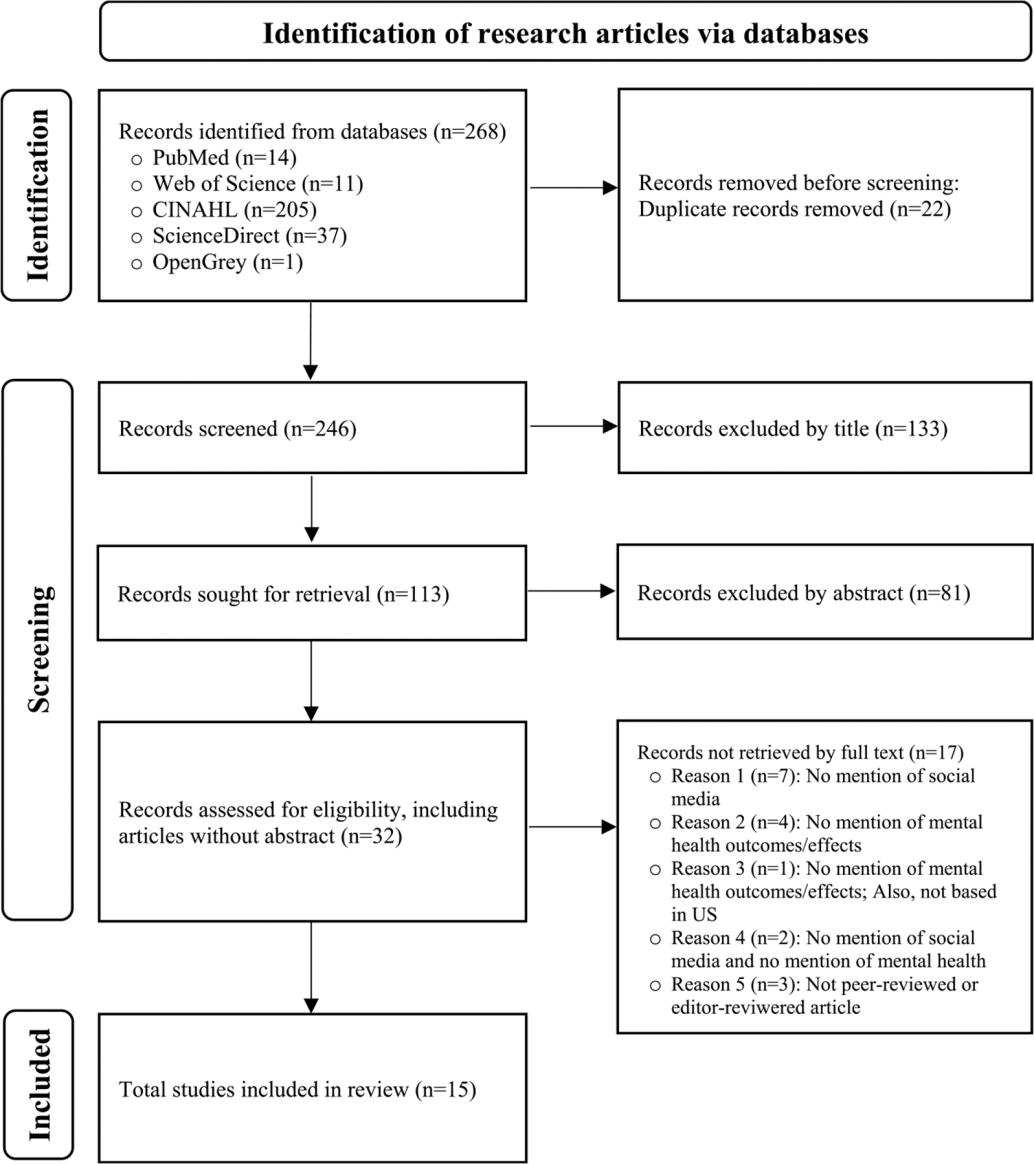
Flow diagram for systematic review.

The detailed process of identifying the relevant research articles is presented in the PRISMA 2020 flow diagram (see Fig. 1). The search and selection process for the systematic review began with identifying 268 records across multiple databases, including PubMed (54 articles), Web of Science (131 articles), CINAHL (20 articles), ScienceDirect (37 articles), and OpenGrey (1 article). From this identification process, 22 duplicate articles were removed, leaving 246 for screening. During the screening process, 133 records were excluded if their titles did not discuss social media, the COVID-19 pandemic, and anti-Asian or racist sentiment online. The abstracts of the remaining 113 articles were assessed, resulting in the exclusion of 81 additional articles. Then, full texts were sought for 32 articles, strictly assessed to see if they fit the inclusion criteria. Of these, 17 articles were excluded due to the criteria of our initial search strategy: 7 did not mention social media (Reason 1), 4 did not mention mental health outcomes or effects (Reason 2), 1 did not mention mental health outcomes/effects and was not based in the United States (Reason 3), 2 mentioned neither social media nor mental health (Reason 4), and 3 were not peer-reviewed or editor-reviewed articles (Reason 5). Ultimately, 15 articles met all of the inclusion criteria and were included in the final review. Excluded articles from the abstract and full-text screening phases, as well as the rationale behind their exclusion, were detailed in Appendix B.

### 3.2. Characteristics of the included studies

Table 1 provides a summary of 15 studies examining anti-Asian hate, discrimination, and sentiment trends during the COVID-19 pandemic. The included studies were published between 2020 and 2024, including recent analyses, such as Nguyen et al. (2024) and Ng and Indran (2024). Sample sizes ranged from 100 TikTok videos (Jacques et al., 2023) to over 55 million tweets (Nguyen et al., 2024). These studies utilized diverse methodologies, including machine learning models (Hswen et al., 2021; Nguyen et al., 2020), qualitative content analysis through thematic analysis of tweets and videos (Jacques et al., 2023; Nguyen et al., 2020), spatial analysis to identify hate clusters geographically (Hohl et al., 2022), and surveys examining mental health impacts and social media use (Pan et al., 2021; C. Yang et al., 2020). Findings highlighted a sharp increase in anti-Asian sentiment early in the pandemic, driven by stigmatizing terms like “Chinese Virus” (Darling-Hammond et al., 2020; Hswen et al., 2021), while counter-hate messaging and resilience narratives emerged on platforms like TikTok (Jacques et al., 2023). Studies also noted the link between social media sentiment and hate crimes, with a 1% point increase in negative sentiment correlating with a 24% rise in anti-Asian hate crimes in New York (Wei et al., 2024). These findings underscore the impact of online discourse on public perception and incidents of discrimination.

### 3.3. Quality appraisal of the included studies

All 15 articles were reviewed using the AXIS quality appraisal tool and categorized into three groups: low quality (<70%), medium quality (70%–80%), and high quality (>80%) (Appendix C). The overall quality of the studies was classified as medium, with a mean score of 75%. Two studies were rated as low quality, with scores ranging from 35% to 65%, while the majority (n = 9) were of medium quality, scoring between 70% and 80%. Four studies were classified as high quality, with scores ranging from 85% to 95%, demonstrating strong methodologies, validated measurements, clear statistical presentation, and adequately described results. Ethical approval and participant consent were reported in the high-quality studies.

### 3.4. Findings

The results of the syntheses were based on six groups–Asian populations, COVID-19 pandemic, racism and discrimination, mental health impacts, role of social media, and contextual relevance to the United States–and then were expanded to four themes. This framework provided a clear structure to analyze the experiences of Asian communities during the pandemic, the amplification of hate through social media, its mental health consequences, and the socio-political context shaping these dynamics.

#### 3.4.1. Spikes of racism, discrimination, and anti-Asian sentiment occurred during the COVID-19 pandemic

Asians and Asian Americans in the United States were subjected to significant levels of racial prejudice and discrimination, as well as excessive attention on social media. The Asian community experienced disproportionately higher rates of racial prejudice and discrimination, alongside increased attention on social media (Cho et al., 2020; Jacques et al., 2023; Ng & Indran, 2024; Nguyen et al., 2020; Pan et al., 2021; Wei et al., 2024). Hate crimes against Asian Americans reportedly increased by over 150% in major U.S. cities during 2020 (Nguyen et al., 2020). Although all authors focused on Asians in the U.S., Ng and Indran (2024) particularly examined Asians of older ages, who faced unique challenges such as cultural and language barriers when responding to online hate. Some studies also included Pacific Islanders, broadening the scope to encompass the Asian American and Pacific Islander (AAPI) (Huang et al., 2022; Ng & Indran, 2024; Wei et al., 2024; Wheeler et al., 2023). Online hate’s negative portrayal contributed to the reinforcement of harmful stereotypes, framing Asian Americans as “foreign” or a “threat,” which perpetuated discriminatory attitudes across different regions in the United States (Cho et al., 2020; Darling-Hammond et al., 2020; Hohl et al., 2022). The national response to the alarmingly high rates of racial prejudice and discrimination included several movements that manifested both online and offline, such as the Stop AAPI Hate movement and the #StopAAPIHate hashtag online (Hohl et al., 2022; Jacques et al., 2023; Wei et al., 2024). The Stop AAPI Hate Reporting Center was similarly established in 2020 in response to this surge, supporting the movement by documenting hate crime incidents and tracking trends in online anti-Asian sentiment, such as the frequency of anti-Asian hashtags (Hohl et al., 2022).

During the COVID-19 pandemic, the United States witnessed a surge in racism, xenophobia, hate crimes, and violence, particularly targeting the Asian community (Jacques et al., 2023; Ng & Indran, 2024; Nguyen et al., 2020; Pan et al., 2021; Wei et al., 2024). The pandemic intensified pre-existing racial biases, with stigmatizing terms like “Chinese virus” and “kungflu” significantly increasing anti-Asian sentiment and correlating with a spike in hate crimes (Hohl et al., 2022; Hswen et al., 2021; Wei et al., 2024). Hswen et al. (2021) noted a 250% increase in anti-Asian hate crimes in 2020 compared to 2019. Historical or social crises, such as the COVID-19 pandemic, often trigger distinct social media trends that amplify societal responses (Nguyen et al., 2024). While anti-Asian sentiment had been declining from 2007 to 2020, the onset of the pandemic reversed this trend, fueling a sharp rise in prejudice and discrimination (Darling-Hammond et al., 2020). Public figures and conservative media played a significant role in spreading this stigmatizing language, shaping public perceptions, and intensifying discrimination (Cho et al., 2020; Hswen et al., 2021; Nguyen et al., 2020; Wei et al., 2024). Hohl et al. (2022) attributed the surge in anti-Asian hate to the “Yellow Peril ideology,” which portrayed Asians as a threat to Western and U.S. culture. While this xenophobic ideology may not have been universally supported, the data highlighting the rise in anti-Asian hate and sentiment was alarming and demanded attention. Between March and May 2020, 1843 hate incidents were reported to the Stop AAPI Hate Reporting Center (Hohl et al., 2022). The establishment of the Stop AAPI Hate Reporting Center reflected the concerning frequency of such incidents. Additionally, the COVID-19 Hate Crimes Act was instituted in 2021 in response to the growing violence (Wei et al., 2024). This pattern of blaming or perceiving racially minoritized groups as threats was not new; similar trends were observed during the 2003 SARS pandemic and the smallpox epidemic (ElTohamy et al., 2024). The scapegoating of marginalized groups for national security and health crises had a long history and persist, evident in this recent pandemic (ElTohamy et al., 2024).

#### 3.4.2. Geographic and temporal patterns of anti-Asian sentiment in social media and real-world incidents

Five articles discussed the association between social media content in particular areas or at a certain time, correlated with the amount of reported anti-Asian hate incidents, or if trends online capture the general attitude of the public (Hohl et al., 2022; Jacques et al., 2023; Ng & Indran, 2024; Nguyen et al., 2024; Wei et al., 2024). Geographic variations of anti-Asian hate incidents were observed across both urban and rural areas. Hswen et al. (2021) and Hohl et al. (2022) revealed that anti-Asian hate tweets during the COVID-19 pandemic were not confined to urban areas but were also prevalent in rural regions. For example, in regions like Ross County, Ohio, where diversity is limited and nationalist sentiment is stronger, hate crimes were particularly prevalent (Hswen et al., 2021). Demographic and ideological differences further influenced the intensity and spread of discrimination (Alexander et al., 2020; Hswen et al., 2021). The spread of Anti-Asian hate incidents during the COVID-19 pandemic reflects a range of geographic, temporal, and socio-cultural dynamics. This geographic distribution underscores the influence of local socio-political and cultural contexts on the prevalence of hate speech. Areas with limited diversity, polarized media consumption, or strong nationalist sentiment may have been more susceptible to xenophobic narratives, such as referring to COVID-19 as the “Chinese virus.” These findings illustrate that online hate speech mirrors broader societal biases and is amplified by digital platforms, transcending physical boundaries. This reinforces the need for targeted interventions addressing both online behavior and underlying regional factors. Hate incidents peaked during the early stages of the pandemic, reflecting a direct correlation between misinformation and hate speech amplification (Hohl et al., 2022). These findings also suggest the need to consider physical location when supporting Asians and Asian Americans, that is, communities may be more vulnerable in some locations than others. Furthermore, by monitoring such sentiment by geospatial data, we may also be able to predict 1) Asian and Asian Americans’ need for social support and 2) potential to identify concerning levels of anti-Asian sentiment, which requires an appropriate intervention.

#### 3.4.3. Association between racial prejudice and discrimination and mental health outcomes

Instances of hate based on race can manifest in various ways, and in the articles reviewed, 11 (Darling-Hammond et al., 2020; Hohl et al., 2022; Hswen et al., 2021; Huang et al., 2022; Jacques et al., 2023; Ng and Indran, 2024; Nguyen et al., 2020, 2024; Pan et al., 2021; Wheeler et al., 2023; Yang et al., 2020) focused on discrimination in the form of hate speech or anti-Asian discourse online, one (Chen et al., 2022) focused on racial discrimination experienced both online and offline, one (Wei et al., 2024) focused on reported hate crimes and violence, one (ElTohamy et al., 2024) focused on vicarious racial discrimination, and one (Cho et al., 2021) focused on stigmatization. Racial discrimination can be defined differently, though it is generally agreed upon to refer to any unequal treatment or perception an individual receives based on their race that is associated with adverse health outcomes (Darling-Hammond et al., 2020; ElTohamy et al., 2024; Pan et al., 2021). Racial prejudice, discrimination and bias have been linked to negative physical and mental health outcomes (Darling-Hammond et al., 2020; ElTohamy et al., 2024; Jacques et al., 2023; Ng & Indran, 2024; Nguyen et al., 2020; Pan et al., 2021; Wheeler et al., 2023; C. Yang et al., 2020). A key contributor to these outcomes is the sense of marginalization and lack of support, both online and offline, which can exacerbate feelings of isolation and vulnerability. This is particularly concerning, as having a strong support system is essential for maintaining health and well-being (Ng & Indran, 2024; C. Yang et al., 2020). Ng and Indran (2024) suggested that these effects were particularly harmful for older Asian Americans, as racism exacerbated health issues associated with aging, making them more susceptible to negative health outcomes. Since social support was critical to the mental and physical health of older adults, instances of racial discrimination were especially detrimental to their perceived social support, highlighting the importance of considering this factor (Ng & Indran, 2024). Experiences of racial discrimination have been associated with mental health conditions such as anxiety, depression, chronic stress, and race-based traumatic stress, underscoring the profound psychological toll of systemic and interpersonal racism (ElTohamy et al., 2024; Pan et al., 2021). While the impacts of racist sentiment on social media on mental health remained unclear, Darling-Hammond (2020) suggested the theories of “media effects” to help explain the full extent of these impacts. Media effects, or changes in behaviors and attitudes resulting from media use, negatively influenced public attitudes about Asian Americans, thereby exacerbating perceived social support (Darling-Hammond et al., 2020).

#### 3.4.4. Social media as a tool of spreading anti-Asian sentiment, as well as instances of counter-hate or social support

There was a common theme of an increase in counter-hate and messages of support for the Asian American community following or in response to news coverage of real-life incidents of hate, such as abuse and physical attacks. Social media exponentially increased the spread of anti-Asian sentiment due to its limited supervision and regulations and censorship of content, regardless of its potential harm (Huang et al., 2022; Jacques et al., 2023; Nguyen et al., 2024). Online trends, to some extent, reflect social attitudes and how communities view and interact with one another (Nguyen et al., 2024). When significant events occur, such as the COVID-19 pandemic and the accompanying rise in anti-Asian hate crimes, social media content and trends shift accordingly (Darling-Hammond et al., 2020; Jacques et al., 2023; Ng & Indran, 2024; Nguyen et al., 2024; Wei et al., 2024). Studies have noted the frequency of anti-Asian sentiment on social media by analyzing the volume of tweets containing such sentiment and identifying tweets that used anti-Asian keywords and hashtags, such as “#chinesevirus” or “#kungflu (Darling-Hammond et al., 2020; Hohl et al., 2022; Pan et al., 2021; Wheeler et al., 2023).

On the other hand, alongside discussion about anti-Asian hate, five groups of authors also examined instances of counter-hate or social support in response to the increase in hate (Jacques et al., 2023; Ng & Indran, 2024; Nguyen et al., 2020; Wheeler et al., 2023; C. Yang et al., 2020). They all found that social media functions as a versatile platform for communities to both oppose and support one another. Wheeler et al. (2023) reported that trends of public support may often stem from “hashtag activism,” driven by social media engagement rather than deeper, more genuine motivations, such as moral conviction or ethical principles. They compared the timelines of the frequency of anti-Asian tweets to counter-hate tweets (e.g. #washthehate) and found that anti-Asian tweets peaked in the middle of the timeline, while counter-hate were most popular towards the end. While social media can be a tool to spread hate, it can also be a platform to spread hope and support (Jacques et al., 2023; Ng & Indran, 2024; Nguyen et al., 2020). Nguyen et al. (2020), in particular, identified several common themes in their qualitative content analysis of tweets. These included “racism,” “blame,” and “misinformation,” themes of “call to action” and “anti--racism.” Yang et al. (2020) specifically reported that certain engagement methods, such as posting and commenting, were associated with higher perceived social support, indicating that social media can serve as a tool to affirm that individuals are not alone.

## 4. Discussion

Through this systematic literature review, we provided a summary and review of common themes in recent literature about anti-Asian sentiment online in the context of the COVID-19 pandemic and its implications for public health and safety in the Asian and Asian American communities. We also analyzed previous studies and discussions on the mental health impacts of discrimination, taking into account how the social media platform and the context of a global pandemic may contribute to a community’s mental health and public safety. The necessity for this research can be summarized in three main points. 1) The fast growing Asian/Asian American populations are an integral part of communities that are in need of attention. Given the historical marginalization and discrimination of Asians and Asian Americans, as well as the recent surge in concern caused by the COVID-19 pandemic, which has further exacerbated racism and discrimination and the prevalence of mental health symptoms, it is imperative to provide support for their mental health. 2) The COVID-19 pandemic resulted in an increase in mental health symptoms, including anxiety, depression, and psychological distress. This can be attributed to the increases in anti-Asian sentiment or racism that took place. Race-based experiences and their associated mental health outcomes, such as anxiety, have been investigated to some extent; however, this area can benefit from further exploration through the platform of social media. 3) The health and safety implications of racist content shared online are still largely unknown, despite the fact that social media is not uncommon for it to be used as a platform for negative sentiment. Given the increasing prevalence of social media in everyday life, it is critical to investigate a) how anti-Asian sentiment is disseminated online and b) how specific content presented online may impact their users in the form of implicit biases, behaviors, and well-being, as prior research on the topic is limited.

### 4.1. Anti-Asian sentiment through social media and health outcomes

The objective of this study was to assess the impact of anti-Asian sentiment on social media on the mental and emotional health and well-being of Asians and Asian Americans during the COVID-19 pandemic. Across the reviewed literature, there was consensus on the negative physical and mental health outcomes associated with racial discrimination and prejudice. However, whether online hate speech and anti-Asian sentiment specifically contribute to these outcomes remains debated, highlighting the need for further research. Social media platforms, such as Twitter, TikTok, Instagram, and Reddit, serve as spaces where anti-Asian sentiment is not only visible but can also escalate from passive hate speech to active harassment through features like comments and private messaging (Costello et al., 2023; Emergency Medicine News, 2020; Jacques et al., 2023; Nguyen et al., 2024; C. Yang et al., 2020). While social media itself is linked to negative health outcomes, including heightened risks of depression and anxiety, exposure to hate content further exacerbates these concerns (Pan et al., 2021). Additionally, trends in hate language on social media often follow significant events such as hate crimes, political rhetoric involving anti-Asian language, and global crises like the COVID-19 pandemic. These patterns underscore the critical need to monitor the influence of such events on social media discourse and their potential impact on the well-being of affected communities (Cao et al., 2022; Hswen, 2022).

Social media, as a widely used communication channel, can amplify voices of support and community resilience while also serving as a powerful tool for spreading hate and harmful narratives, as well as a source of vicarious discrimination (Huang et al., 2022; Jacques et al., 2023; Nguyen et al., 2024). This review, along with other relevant literature, suggests that racism on an online platform carries a high risk of harm. For example, Cano et al. (2021) examined this association among the Hispanic community, finding that higher exposure to social media discrimination was linked to higher symptoms of depression and generalized anxiety. Cano et al. (2021) concluded that online ethnic discrimination is a sociocultural stressor that is largely underappreciated and critical, particularly as various racial and ethnic groups experience varying trends in negative sentiment (Nguyen et al., 2020, 2024). This gives way for future researchers to explore how social media can be used as a mediating factor in the relationship between anti-Asian sentiment and mental health outcomes. Yang et al. (2020) explored how experiences of discrimination impact social media use and well-being. They found that social media engagement in the form of browsing was associated with higher levels of worry about discrimination. In turn, Pan et al. (2021) found no association between worry about discrimination and depression. Social media private messaging and posting or commenting were linked to improved subjective well-being due to increased perceived social support, suggesting that social media serves as a more beneficial form of interpersonal communication compared to its role as a source of entertainment and information, such as browsing. These contrasting findings suggest that only specific forms of social media engagement are associated with negative health outcomes, and further research should be mindful of the nuanced nature of social media engagement.

### 4.2. Racist sentiment on social media may influence implicit biases and racist behavior through social media algorithms and user behavior

The structure of social media should also be investigated more closely, as it can influence different behaviors that may have different psychological health and safety for users. Zimmer et al. (2019) suggested “echo chambers” and “filter bubbles” to be prevalent on social media and influential on users’ behaviors. “Echo chambers” in the context of social media refers to a situation where only particular ideas and beliefs are shared continuously. A “filter bubble” refers to a situation in which an algorithm personalizes particular content based on the social media user. Additionally, the content presented to a user is highly dependent on the user’s behavior and social media algorithms reflect and filter the content presented based on that (Zimmer et al., 2019). This may suggest that in the areas of high anti-Asian sentiment online as presented by Nguyen et al. (2024), Hohl et al. (2022), and Wei et al. (2024), “echo chambers” or “filter bubbles” consisting of racist implicit biases or beliefs may also be just as prevalent. This can be a concerning area because such content may lead to vicarious discrimination if it contains racist sentiment, which is linked to increased race-based stress symptoms (ElTohamy et al., 2024) and heightened worry about discrimination (C. Yang et al., 2020). However, this may also explain how social media users found social support as a result of these algorithms, as supportive content, such as the hashtag “#StopAAPIHate,” appeared more prevalent in response to the trends of negative sentiment (Hswen et al., 2021; Jacques et al., 2023; Ng & Indran, 2024; Wheeler et al., 2023; C. Yang et al., 2020). Given these factors, racist sentiment on such a platform may amplify the impacts of racist sentiment in a way that is distinct from in-person discrimination. The difference in modality is critical to explore as social media becomes more prevalent in daily life. Social media trends in the United States are not uniform; their intensity varies by location and shifts temporally around significant events. For instance, Hohl et al. (2022) identified clusters where hateful tweets were 312 times higher in relative risk compared to other areas. Interestingly, their study found no discernible pattern to explain the geographic distribution of these clusters. This unpredictability could be linked to the workings of social media algorithms, which are designed to recommend content and groups tailored to individual users. Such algorithms can create “echo chambers” that amplify certain ideas and, in some cases, escalate these ideas into real-world actions. This phenomenon becomes especially concerning when the content originates from influential figures, such as a president or a senator (Stewart & Beckman, 2023; Wei et al., 2024; Wheeler et al., 2023).

This review’s findings suggest a complex interplay between online and offline hate, where online hate speech may contribute to the escalation of hate crimes, or hate crimes may fuel the prevalence of hate speech online. Social media activity could potentially serve as an indicator of the likelihood of hate incidents occurring offline, highlighting the possibility that monitoring online hate activity may help mitigate hate incidents in the real world. Additionally, discrimination on social media may influence offline hate crimes and discrimination, or online anti-Asian trends may reflect existing patterns of hate and discrimination occurring offline. These interconnected dynamics emphasize the need for further research to better understand the relationship between online hate activity and its real-world consequences, particularly since only one study, as per our search, has examined this particular association (Wei et al., 2024). They investigated the association between anti-Asian sentiment online and the prevalence of hate crimes. While the investigation was conducted on a limited scale in a single city in New York, the positive association observed indicated that this may be a consistent pattern in other cities. Given Hohl et al. (2022) and Nguyen et al. (2024) documented geospatial trends in anti-Asian sentiment, it is possible that hate crimes may also occur in proportion to those locations. However, the predictability of this may differ, as Nguyen et al. (2024) identified a higher proportion of negative sentiment tweets that primarily manifested in other states, whereas Hohl et al. (2022) found no identifiable pattern. They instead identified both clusters in highly populated urban areas and one rural county in Ohio. Online sentiment may not be as innocent as it appears, particularly in the context of racist and discriminatory behavior. In contrast, Alsaad et al. (2018) conducted a study that discovered a negative association between social media use and racist behavior. This finding implies that social media may not have a significant impact on hate crime incidents, assuming that hate crimes and racist behavior are associated and may be influenced by alternative factors. Such research is essential to developing effective strategies to address the escalation of hate crimes influenced by online hate speech and the online manifestations of pre-existing offline discrimination.

### 4.3. Limitations

This systematic literature review had a few limitations. One limitation was the variability in the timeline of the COVID-19 pandemic across studies. Some studies were cross-sectional, some spanned a few months, while others extended over several years. This inconsistency in defining a clear timeline could potentially act as a confounding factor. Another limitation was the ambiguity in determining the directionality of mental health issues. It was unclear whether these issues arose directly as a result of discrimination on social media, from social media use exclusively, or from discrimination alone. Additionally, studies discussing discrimination as a risk factor or exposure were often unclear about whether online discrimination was included. When it was included, the proportion of online to offline instances of discrimination was not specified. Pan et al. (2021) explored the association between depression and three factors: racism-related social media use, experiences of discrimination, and worry about discrimination. Their findings suggest that racism-related social media use should be examined as a distinct variable, rather than being grouped under broader categories like “racism” or “discrimination.”

Another limitation was the inconsistency in defining the target population of Asians and Asian Americans. Some studies explicitly included Pacific Islanders as part of the Asian category (Huang et al., 2022; Ng & Indran, 2024; Wei et al., 2024; Wheeler et al., 2023). This limitation highlights how racial group classifications can be subjective rather than objective, potentially leading to the inaccuracy of generalization, particularly when encompassing diverse subgroups within an Asian and Pacific Islander category.

The unclear directionality in the relationship between social media trends, anti-Asian sentiment, racial bias, and real-life hate incidents (e. g., hate crimes) posed another limitation. For example, it remains uncertain whether anti-Asian hate crimes were a direct result of an influx of anti-Asian sentiment online or if the online sentiment followed hate crimes and other significant events. Similarly, the directionality between mental health outcomes and anti-Asian hate or online discrimination was unclear. The COVID-19 pandemic itself was a significant source of psychological stress due to various factors, such as financial instability, social isolation, and uncertainty about the future. While it is evident that mental health concerns escalated during COVID-19, and social media activity and anti-Asian sentiment also rose during this period, disentangling these relationships is challenging. Ongoing research and discourse on this topic are essential to fully assess the extent to which social media influences individual behaviors and health outcomes, especially given its constantly evolving nature.

### 4.4. Implications

The authors from the selected articles collectively highlighted that online discrimination could lead to harmful outcomes by fostering a hostile environment that normalizes prejudice and invites further acts of discrimination. Their research identifies several strategies that public health officials can implement to mitigate such exposures and their negative consequences. One proposed strategy involves using surveillance on social media platforms to monitor population sentiments and identify community needs. For example, Facebook mobility monitoring in Taiwan was effectively employed during the COVID-19 pandemic to track social distancing and travel patterns, aiding in the understanding of transmission trends (Huang et al., 2022). Similarly, surveillance can serve as an early warning system to identify indicators of potential health crises, such as symptoms predicting illnesses (Hswen et al., 2021; Huang et al., 2022; Wei et al., 2024).

Another key recommendation is the incorporation of culturally competent language and discourse in public communication, particularly by news outlets, political leaders, and public health officials. Additionally, in order to properly enforce such a critical practice, there should also be accountability measures in place if language occurs and persists. Training in cultural sensitivity can help prevent the encouragement and normalization of racial prejudice (Benner et al., 2024; Chen et al., 2022; Darling-Hammond et al., 2020; ElTohamy et al., 2024; Hswen et al., 2021; Jongsma et al., 2021; Nguyen et al., 2024; Wei et al., 2024). For instance, the use of terms like “Chinese virus” by influential figures, such as the former President on Twitter, was shown to normalize hate-based language and exacerbate discrimination (Darling-Hammond et al., 2020; Hswen et al., 2021). The previous studies also highlighted the mental health consequences of such discrimination, with seven studies emphasizing the urgent need for more research in this area (Darling-Hammond et al., 2020; ElTohamy et al., 2024; Hohl et al., 2022; Hswen et al., 2021; Nguyen et al., 2020, 2024; Wheeler et al., 2023). In addition to mental health concerns, high prevalence of anti-Asian sentiment may also have other health implications such as hesitance in COVID testing and disclosure to health providers due to stigma (Chen et al., 2022). Additionally, several studies called for expanded mental health services and programs to help individuals cope with experiences of discrimination (Cho et al., 2020; ElTohamy et al., 2024; Hohl et al., 2022; Pan et al., 2021; Wei et al., 2024). They advocated for broader cultural shifts, including increased awareness of the effects of social media, the historical and ongoing impact of racial discrimination, and the dangers of ageist ideologies. Such shifts are essential for fostering a more inclusive society and mitigating the harmful effects of discrimination (Chen et al., 2022; Hohl et al., 2022; Jacques et al., 2023; Ng & Indran, 2024; Nguyen et al., 2020; C. Yang et al., 2020).

Xenophobia, discrimination, and anti-Asian sentiment have deep historical roots, and their persistence on digital platforms highlights the dual role of social media. Research can further investigate how social media algorithms play a role. Individual social media users can only do so much, such as self-monitoring and reporting harmful content, but social media platforms should be held accountable if racist sentiment is actively displayed to individuals who are most vulnerable to it, especially if there is already research supporting its negative outcomes. Huang et al. (2022) discussed how detection of hate and racism existed prior to the pandemic, even in other languages other than English. However, the extent to which social media platforms and officials are using these algorithms to take action, such as responding to reported discriminatory and hateful content, rather than allowing the promotion of online activity for profit, should be brought to their attention in order to hold them accountable and remind them of their level of responsibilities. Additionally, Twitter was the primary social media platform investigated. Other social media platforms should also be investigated, such as Instagram, TikTok, and YouTube, since their users include primarily a younger demographic.

The lack of adequate monitoring and regulation underscores the need for policymakers and public health officials to leverage social media trends for assessing community concerns and addressing harmful behaviors. Proactively monitoring online discourse can not only help identify emerging threats but also inform public health campaigns and resource allocation to mitigate the negative impacts of discrimination. Wei et al. (2024) discovered a positive relationship between anti-Asian sentiment on Twitter and levels of anti-Asian hate crimes in New York City, which allows researchers to investigate the nature of hate crimes in general. Wei et al. (2024), Hohl et al. (2022), and Huang et al. (2022) argued for increased monitoring. Furthermore, given that most social media platforms cross national borders, it may be beneficial for various governments and public health institutions to collaborate and address the issue of social media as a conduit for racist sentiment and its impact on mental health (Huang et al., 2022). Upon further investigation into the association of hate crimes and online racist sentiment, officials could potentially identify warning signs and increase support and vigilance for vulnerable communities.

By fostering culturally competent communication and promoting responsible social media practices, these efforts can transform platforms into spaces of inclusion and support rather than division and harm. Future research should continue to examine the causal relationship and directionality between online anti-Asian hate speech and mental health outcomes to better understand its direct and indirect effects. Additionally, studies should specifically examine the impact of social media activity on offline hate crime in Asian American communities and explore culturally tailored interventions to mitigate discrimination and foster community resilience.

## Supplementary Material

1

2

3

## Figures and Tables

**Table 1:** Characteristics of selected studies.

Authors, Year	Objective	Study Design/Method	Data Collection	Sample Characteristics	Main Findings
Ng and Indran (2024)	To explore the discourse around older Asian Americans during the COVID-19 pandemic, in relation to the spike in anti-Asian sentiments.	Cross-sectional, descriptive study; Tweets retrieved through two search queries, 1) Tweets with the #ProtectOurElders, 2) Tweets with age-based terms, like “elderly,” and either the #StopAAPIHate or #StopAsianHate, from Jan 1, 2020, to Aug 1, 2023. After exclusion criteria, repeats and contextually irrelevant tweets, inductive and deductive approaches informed qualitative content analysis.	English tweets collected using version 2 of Twitter’s Application Programming Interface (API) through 2 search queries of terms related to age and anti-Asian hate; Excluded “retweets” to avoid any duplications.	N = 994 tweets containing relevant hashtags and terms focusing on older Asian Americans during the COVID-19 pandemic period.	Four major themes were found: 1) framing older Asian Americans as “vulnerable and in need of protection,” 2) as “heroic and resilient,” 3) framing them as “immigrants who have made selfless contributions and sacrifices,” and 4) as “worthy of honor,” describing the response on Twitter to the rise in violence toward this population during COVID-19. Support for community fluctuates with time.
Jacques et al. (2023)	To examine the discourse and messaging that offers support for communities and condemnation of Asian hate on TikTok.	Cross-sectional, descriptive study; Content with the #StopAsianHate tag were included in this study, as it was the most used hashtag relating to the Asian community on TikTok. Videos were watched by reviewers, collecting performance metrics (views, likes, shares, followers). Ran independent one-tailed t-tests and kept descriptive analysis on MS Excel.	First 100 English videos to result from search query “#StopAsianHate” through TikTok’s “discover” feature in Jan 2022 were collected and included in analysis; Excluded 25 unrelated videos.	N = 100 English TikTok videos addressing anti-Asian hate, focusing on Asians and Asian Americans during the pandemic.	11 themes were found, most notably: Asian Abuse/Attack (includes physical & verbal), awareness on Asian hate & hate crime, stop hate messaging, mention of COVID/virus is hate, fight back/defending, stereotypes or microaggressions, positive Asian representation, humor/Asian jokes, and empathy, from highest to lowest performance.
Nguyen et al. (2024)	To provide practical guidance on using social media data mining for measuring public sentiment.	Analytical, observational study over a long study period; Data collection from Twitter from Jan 1, 2011, to Dec 31, 2021, using one or more 90 race-related keywords and utilized Support Vector Machine (SVM), a supervised machine learning model for binary sentiment analysis; Restricted analysis to tweets using one or more of 90 race-related keywords.	Random sampling of English tweets from Twitter’s API from the U.S. that contained keywords relating to racially minoritized groups in the U.S. during the 10-year study period.	N = 55,844,310 U.S.-based tweets from 3,699,646 users based on keywords related to racially minoritized groups in the U.S.	Proportion of tweets with negative sentiment, referencing racial groups increased at the county, state, and national levels, with a 16.5% increase at national level during study period. Analysis revealed unique patterns reflecting and aligning with historical events specific to each racial group, such as the emergence of COVID-19 and anti-Asian sentiment.
Darling-Hammond et al. (2020)	To evaluate if and how racially charged media coverage, particularly use of stigmatizing terms such as “Chinese virus,” influenced bias against Asian Americans.	Cross-sectional analysis; Data from survey answers from non-Asian respondents of the Project Implicit “Asian IAT” were included and analyzed, data spanning from Jan 1, 2007, to Mar 31, 2020, split into two data sets: 1) Jan 2007 to Feb 2020 and 2) after Feb 11, 2020, until the latest date in Project Implicit data, Mar 31, 2020. Analyses focused on a period from Feb 11, 2020, to Mar 31, 2020, when the media’s tone shifted to stigmatizing language related to COVID-19 and its origin.	Data collection from respondents of the Project Implicit “Asian IAT” from 2007 to 2020. Data categorized into two sets, before and after Feb 11, 2020 (WHO guided media outlets to avoid stigmatizing language when referring to COVID-19) until Mar 31, 2020.	N = 339,063 non-Asian respondents in the U.S. of the “Asian IAT.” Implicit Americanness Bias measured association of “Asian American” with American or foreign symbols.	A significant reversal in bias trends occurred after Mar 8, 2020, correlating with a spike in the use of terms like “Chinese Virus” in conservative media, and increase in “Implicit Americanness Bias” suggested Asian Americans were seen as less American. The use of stigmatizing language increased subconscious beliefs that Asian Americans are “perpetual foreigners,” thus, providing guidance for the media. The trend was found to be more pronounced among conservative individuals.
Wheeler et al. (2023)	To investigate how anti-Asian hate and counter-hate messages on Twitter varied over the first 16 months of the COVID-19 pandemic.	Sensitive analysis; Queried and collected tweets containing anti-Asian or counter-hate hashtags and terms. Latent growth curve modeling to analyze temporal changes in anti-Asian and counter-hate keywords over 16 months (Jan 30, 2020, to Apr 30, 2021). Random subset of 1000 users analyzed to evaluate individual changes in keyword usage over the data collection period.	Collected publicly available tweets through Twitter’s API that were posted between Jan 30, 2020, when WHO declared COVID-19 a global health issue, and Apr 30, 2021, the last day before AAPI Heritage Month.	N = 13,008,053 tweets from 3,298,940 users who posted tweets containing either anti-Asian or counter-hate keywords during study period.	Anti-Asian keywords peaked early in the pandemic and followed a curvilinear pattern of decline, while counter-hate messages increased linearly, specifically after violent incidents against Asian Americans. Total number of anti-Asian keywords ranged from 0 to 46 in a given month (highest occurrence in Mar 2020); Total number of counter-hate keywords ranged from 0 to 60 (highest occurrence in Mar 2020).
Chen et al. (2022)	To explore the impacts of within-community discrimination of Asian Americans relating to COVID-19.	Descriptive and qualitative exploration based on the narratives and existing literature reflecting the experiences of Asian Americans in the context of COVID-19, focusing on the dynamics of within-community discrimination, stigma, social and cultural influences and the implications on health and well-being of it on these communities.	N/A; Focused on Asian American experiences, within-community, and social network dynamics. Sourced from existing reports and studies finding implications on wellbeing and public health.	Examines Asian Americans focusing on experiences of stigma and discrimination related within social networks.	Within-community discrimination for Asian Americans often stems from stigma, beliefs and fear relating to COVID-19 and can come in the form of exclusion or isolation which can negatively impact COVID-19 testing, diagnosis, and prevention behaviors. External influences like social networks and media in Asia contribute to these dynamics.
Hswen et al. (2021)	To examine the extent to which the phrases, “COVID-19” and “Chinese virus” were associated with anti-Asian sentiments.	Cross-sectional and analytical study; Data collection from Twitter’s API, including the hashtags “#covid19” or “chinesevirus.” Analyzed tweets from Mar 9, 2020, to Mar 23, 2020, aligning with the week before and after President Donald J. Trump’s tweet containing the phrase “Chinese Virus” Conducted a hashtag and a tweet analysis.	Tweets from Twitter’s API including either #covid19 or #chinesevirus and posted during study period. Excluded tweets in hashtag analysis that met those criteria but contained no other hashtags.	N = 668,597 tweets and N = 1,273,141 English hashtags from March 9–23, 2020, containing either #covid19 or #chinesevirus.	One fifth (19.7%) of tweets with #covid19 showed anti-Asian sentiment, whereas half (50.4%) of the hashtags with #chinesevirus showed anti-Asian sentiment. After Mar 16, 2020, there was a significantly greater increase in anti-Asian hashtags associated with #chinesevirus than #covid19. Both hashtags increased in prevalence after Trump’s Tweet.
Yang et al. (2020)	To explore the association of subjective well-being and experience of discrimination through nonpassive social media use, and thus, worry about discrimination.	Cross-sectional study; Utilized an online survey. Participants reported experiences of discrimination, social media use, perceived social support, worry about discrimination, and subjective well-being based on their experiences since COVID-19 emerged. Exploratory factor analysis (EFA) of social media use scale, then principal axis factoring with direct oblimin rotation, then, scree plot and the principle of eigenvalues and finally examined the hypothesized path model.	Participants recruited from Amazon Mechanical Turk in May 2020; Data from scales in distributed online surveys to measure five variables: Experience of discrimination, social media use, perceived social support, worry about discrimination, and subjective well-being.	N = 242 participants considering their experience in the context of the COVID-19 pandemic; Identified as Asian or Asian American and resided in the U.S.	Four indirect relationships were found: experience of discrimination was related to better subjective well-being through (a) more private messaging, thus, more perceived social support, (b) more posting/commenting, thus, more perceived social support, (c) more posting/commenting, thus, less worry about discrimination, and (d) experience of discrimination was associated with poorer subjective well-being through more browsing and thus, more worry about discrimination.
ElTohamy et al. (2024)	To evaluate the impact of vicarious discrimination and to examine the association between vicarious discrimination and race-based symptoms.	Cross-sectional descriptive analysis of data from COVID-19 Adult Resilience Experiences Study (CARES), a series of three cross-sectional surveys: 1) Apr 13, 2020 to Aug 31, 2020, 2) Sep 21, 2020 to Mar 15, 2021, 3) After Atlanta shootings. Performed linear regression analysis and a multiple linear regression model to assess relationship between race-based stress symptoms and vicarious discrimination. Descriptive analysis of measures using STATA v17.	From online survey data from COVID-19 Adult Resilience Experiences Study (CARES), which assessed sociodemographic characteristics and key psychometric scales of young adults in the U.S. from Apr 2020 to May 2021. Data categorized into three waves.	N = 135 Asian American young adult (18–30 years old) respondents residing or studying in the U.S., and categorized into three waves based on different stages of the pandemic.	Direct discrimination and vicarious discrimination, as in, witnessing discrimination against a member’s own racial or ethnic group were found to be associated with increased levels of race-based stress symptoms. Common sources of vicarious discrimination were from hearing or seeing instances on the news, social media, or online, thus, the increased use of anti-Asian rhetoric and sentiment on those sources can result in increased race-based stress symptoms for the Asian community.
Pan et al. (2021)	To study the relationship between three discrimination-related variables and depression during COVID-19 among Asians in the U.S.	Cross-sectional survey study; Online survey distributed through Amazon Mechanical Turk (MTurk) in mid-May 2020; Confirmatory factor analysis in Mplus (version 7; Muthén & Muthén) considering experience of and worry about discrimination, social media use, and depression as latent variables. Used SPSS software (version 26.0; IBM Corp) to perform a 3-step hierarchical regression analysis.	Web-based, cross-sectional survey created on Qualtrics and distributed to participants recruited through MTurk in mid-May 2020. Eligible participants included those belonging to Asian ethnic groups, residing in the U.S.	N = 209 Asians in the U. S.; Ages ranged from 18 to 73 years; Participants identified as Chinese (35.41%) or not Chinese (64.59%).	Social media use involving experiences of discrimination and relating to racism was found to be positively associated with depressive symptoms. Worry about discrimination was not associated with depression. Suggests and supports the negative impact of discrimination and racism-related social media on mental health and wellbeing among Asians in the U.S. during the COVID-19 pandemic.
Nguyen et al. (2020)	To analyze shifts in anti-Asian sentiment and describe themes of discussions on Twitter before and after the emergence of COVID-19.	Analytic sensitive study; Conducted a mixed-methods approach, utilizing sentiment analysis and qualitative analysis. Collected tweets from a random 1% sample and applied a supervised machine learning model, Support Vector Machine (SVM) to perform sentiment analysis. A subset of 3300 tweets underwent qualitative content analysis to explore themes like racism, blame, anti-racism, and daily life.	English U.S.-based tweets collected through Twitter’s API including one or more words from a list of 518 race-related terms and one or more terms from a list of 75 COVID-19 terms with U.S. location data during Nov 2019 to Jun 2020.	N = 3,377,295 tweets from 521,161 Twitter users from the U.S. during study period. Tweets contained terms related to race and COVID-19.	The proportion of negative tweets referencing Asians increased by 68.4 %, while negative tweets referencing other racial/ethnic groups remained relatively constant through time, though were higher in frequency. Most common themes were daily life impacts from the pandemic, racism and blame (20%), and anti-racism (20%). Across all categories 18% of tweets mentioned President Trump.
Wei et al. (2024)	To investigate the association between Twitter sentiment and anti-Asian hate crimes in New York City before and during COVID-19.	Analytical, sensitive analysis; Collected tweets from New York State and determined positive or negative sentiment with an (SVM). Analyzed anti-Asian hate crime data, linked to police precinct-level geographic data and used negative binomial models to assess relationship between sentiment and crime rates. Adjusted for variables (ex/unemployment and emergence of COVID-19).	Tweets collected from a random 1 % sample through Twitter’s API from 2019 to 2022. Tweets included one or more of selected Asian-related keywords. Hate crime data from New York City Police Department.	N = 868,012 tweets referencing Asians; Filtered for keywords from users in New York State and anti-Asian hate crime data in NYC.	Found a 1% increase in negative sentiment was associated with a 24% increase in anti-Asian hate crimes in the same month. Positive sentiment was associated with a decrease in hate crimes, but the relationship was not significant after adjusting for unemployment and COVID-19. Higher negative sentiment was associated with more hate crimes in the same month when targeting the Asian community.
Huang et al. (2022)	To summarize the progress of social media data mining during the COVID-19 pandemic to aid public health emergency processes.	Narrative review of existing social media mining studies and publicly available datasets discussing efforts related to the COVID-19 crises. Identified and assessed various social media mining challenges and potential positive impacts if utilized appropriately. Explored content across various social media platforms like Twitter, Sina, Weibo, Facebook, YouTube, Instagram, and Reddit.	N/A (qualitative and review-based analysis); Reviewed content included social media mining efforts and public datasets sourced from pivotal papers known to the subject experts, rather than from a systematic query.	Analysis focused on different types of data across multiple social media platforms Twitter, Sina, Weibo, Facebook, Instagram, and Reddit.	Six domains of social media data mining efforts were identified: Public health and crisis responses benefited from social media insights, while challenges such as biased sampling, multilingual data complexities, posting incentives, positioning accuracy, and sentiment analysis inconsistencies were highlighted. Implications for utilizing data mining for the benefit of public health were provided.
Hohl et al. (2022)	To illustrate and assess the spatiotemporal distribution of geolocated tweets that contain anti-Asian hate language in the U.S. during the early stage of the COVID-19 pandemic.	Cross-sectional study; Used a data set of geolocated tweets from Nov 2019 to May 2020 that match a list of COVID-19-related keywords and further classified based on the presence of anti-Asian hate terms; Final sample assigned to U.S. counties for spatial analysis. Identified geographical clusters using space-time scan statistic (STSS) with Bernoulli model, that finds the most likely cluster of hateful tweets. Visualized distribution as a choropleth map of the relative risk with circular clusters identified by STSS.	Purchased a data set of geolocated tweets through Twitter’s API; Inclusion of Tweets based on if matched a list of keywords that represent anti-Asian hate in general, as well as context-specific to COVID-19; Tweets with imprecise location information (only state-level) excluded.	N = 4,234,694 English geolocated tweets in the contiguous U.S.; U.S.-based Twitter users containing keywords related to COVID-19 and hate toward Asians and Asian Americans.	Anti-Asian hate language surged between Jan and Mar 2020. Found 15 clusters of hate across the contiguous U. S. The strongest cluster consisted of a single county (Ross County, Ohio), where proportion of hateful tweets was 312.13 times higher than the rest of the country. Significant clusters were in rural areas and areas that have high-population densities. Anti-Asian hate on Twitter exhibits a significantly clustered spatiotemporal distribution. Clusters varied in size, duration, strength, and location with no identifiable pattern.
Cho et al. (2020)	To investigate factors associated with stigmatization of Asian people during COVID-19 in the U.S. and explore the factors that can affect stigmatization.	Cross-sectional study; National sample survey conducted online between May 11 and May 19, 2020. Nine items were assessed. The outcome variable, stigmatization, were measured to include two dimensions, responsibility and persons as risk. Hierarchical regression analyses were performed using a three-step approach, using statistical software R version 3.5.0. Developed and tested a conceptual model aimed to explain the stigmatization of Asian communities.	Nationally representative sample of U.S. adults from online survey through Dynata’s national panel during study period; measures of all items were on a Likert scale unless noted otherwise; Items were assessed like “self-efficacy,” “media use” and “facial prejudice.”	N = 842 U.S. adults, 18 and older (Mean age was 51.5 years). Racial composition included white, Black, Hispanic, and mixed race. Asian respondents were excluded.	Racial prejudice, maladaptive coping, and biased media use all impacted stigmatization. Racial prejudice, comprising of stereotypical beliefs and emotion toward Asian Americans, was a stronger predictor of stigmatization than maladaptive coping or biased media use. Fear concerning the ongoing COVID-19 situation and the use of social media and partisan cable TV also predicted stigmatization. More indirect contacts with Asians through the media predicted less stigmatization.

## Appendix A. Supplementary data

Supplementary data to this article can be found online at https://doi.org/10.1016/j.ssaho.2025.102282.
