# Supplementary material for: Anti-Asian sentiment on social media and mental health among Asian and Asian American populations in the United States during the COVID-19 pandemic: A systematic literature review
Source: Soc Sci Humanit Open. Author manuscript; Available in PMC 2025 Dec 31. (PMC12753001; doi:10.1016/j.ssaho.2025.102282)
Supplement: 1 [file NIHMS2130948-supplement-1.pdf]

Appendix A. Online database search strategy (as of October 8, 2024)

PubMed	Web of Science	CINAHL	ScienceDirect	OpenGrey
("Asian People"[Mesh] OR “Asian”[Mesh] OR “Asian”[tw] OR “Asian American*”[tw] OR “Asian immigrant*”[tw]) AND ("Pandemics"[Mesh] OR “Covid-19”[Mesh]) AND ("Racism"[Mesh] OR "Social Discrimination"[Mesh] OR “Discrimination, Psychological”[Mesh] OR "Prejudice"[Mesh] OR "Social Stigma"[Mesh] OR “xenophobia”[Mesh] OR “anti-Asian”[tw] OR “Hate crime”[tw] OR “Hate Speech”[tw] OR “#StopAAPIHate”[tw] OR “Stop AAPI Hate”[tw]) AND ("Psychiatry and Psychology Category"[Mesh] OR "Mental Health"[Mesh] OR "Depressive Disorder"[Mesh] OR "Depression"[Mesh] OR "Anxiety"[Mesh]) AND ("Social Media"[Mesh] OR “X”[tw] OR “Twitter”[tw] OR “Sentiment Analysis”[tw]) AND ("United States"[Mesh] OR "United States"[tw])	("Asian People" OR “Asian” OR “Asian American*” OR “Asian immigrant*”) AND ("Pandemic*" OR “Covid-19” OR “corona virus” OR “coronavirus”) AND ("Racism" OR "Social Discrimination" OR “Discrimination” OR “racial discrimination” OR "Prejudice" OR “xenophobia” OR “anti-asian hate” OR “anti-asian sentiment” OR “victimization” OR “stigma” OR “stigmatization” OR “anti-Asian” OR “Hate crime” OR “Hate Speech” OR “#StopAAPIHate” OR “Stop AAPI Hate” OR “Social media activism” OR “activism”) AND (“mental wellbeing” OR “quality of life” OR “Mental health distress” OR “Mental health” OR “mental health impact” OR “mental health outcomes” OR “stress”) AND ("Social Media" OR “X” OR “Twitter” OR “Sentiment Analysis”) AND ("United States")	(MH "Asian American" OR "Asian*" OR "Asian Immigrant*") AND (MH "COVID-19" OR "COVID-19 Pandemic*" OR "Pandemic*" OR "coronavirus") AND (MH "Anti-Asian Racism" OR "Racism" OR "Xenophobia" OR MM "Discrimination" OR "racial discrimination" OR “hate crime*" OR "hate" OR "hate speech" OR "prejudice" OR "Stop AAPI Hate" OR "#stopaapihate OR "Victim*") AND ("Psychological" OR "mental health distress" OR MM "mental health" OR "Mental Disorders" OR "mental health" OR "psychological distress" OR "health status" OR "quality of life") AND (MH "Twitter" OR "Sentiment Analysis" OR "X” OR "Social Media") AND (MH “United States”)	(“Asian American” OR “Asian immigrant”) AND (“COVID-19”) AND (“Discrimination” OR “anti-asian”) AND ("mental health") AND (“Sentiment Analysis” OR “Twitter”) AND (“United States”)	(“Asian People” OR “Asian” OR “Asian American*” OR “Asian immigrant*”) AND ("pandemic*" OR "covid-19" OR "Coronavirus") AND (“Social Discrimination” OR “Perceived Discrimination” OR “Psychological Discrimination” OR “hate crime*” OR “hate speech” OR “anti-asian” OR “racism” OR “xenophobia” OR “#StopAAPIHate” or “Stop AAPI Hate” OR “prejudice” OR “racial prejudice” OR “stigma*” OR “victim*”) AND (“mental health distress” OR “mental health”) AND (“Social Media” OR “X” OR “Twitter” OR “Sentiment Analysis”) and (“United States”)
