# Supplementary material for: Anti-Asian sentiment on social media and mental health among Asian and Asian American populations in the United States during the COVID-19 pandemic: A systematic literature review
Source: Soc Sci Humanit Open. Author manuscript; Available in PMC 2025 Dec 31. (PMC12753001; doi:10.1016/j.ssaho.2025.102282)
Supplement: 2 [file NIHMS2130948-supplement-2.pdf]

Appendix B. Excluded articles from abstract and full-text screening phases and reasons for exclusion

Author, Year	Reason for exclusion
**Articles Excluded by Abstract (n=81):**	
Liang et al., 2024	3
Dong et al., 2023	1
Choi 2021	1
Ji & Chen 2023	1
Quach et al., 2021	1
Quarishi 2023	1
De Leon et al., 2023	1
Han et al., 2022	4
Pahl et al., 2023	4
Jeung 2024	1
Le et al., 2020	5
Lantz et al., 2023	4
Kim et al., 2022	4
Wang et al., 2023	4
Woo et al., 2021	4
Nie 2022	1
Nie 2024	4
Yang & Tsai 2023	7
Ogedegbe et al., 2020	4
Dababnah et al., 2021	4
Song et al., 2022	1
Gee et al., 2022	1
Pan et al., 2021	1
Omori et al., 2023	2
Costello et al,. 2023	2
Yang et al., 2022	1
Keum et al., 2023	1
Hsiu-Lan et al., 2021	1
Benner et al., 2024	1
Tirupathi et al., 2020	4
Woo & Jun 2022	1
Cheng et al., 2022	1
Ahn et al., 2022	1
Kim et al., 2022	1
Rahman et al., 2023	1
Ching 2022	1

Sakki & Castrén 2022	6
Zafra & Santhiveeran 2024	1
Magesh et al., 2021	1
Shen et al., 2024	3
Cheng et al., 2022	1
Fan 2023	5
Yang et al., 2023	1
Yu et al., 2020	2
Tao & Fisher 2022	6
Banerjee et al., 2020	6
Lee & Howard 2023	1
Amani et al., 2022	1
Lee 2023	1
Yi et al., 2023	1
Sofia et al., 2023	1
McGarity-Palmer et al., 2023	1
Jinbing et al., 2022	1
Ho et al., 2020	1
Hill et al., 2023	1
Yashadhana et al., 2022	1
Yang et al., 2022	3
Woo-Padoongpatt et al., 2022	4
Goyal et al., 2024	6
Chen et al., 2020	1
Lozano et al., 2022	1
Misra et al., 2020	1
Liu et al., 2024	1
Tao et al., 2024	1
Rivera et al., 2023	1
Lu et al., 2024	1
Dong et al., 2024	1
Villadrich 2021	1
Lozano et al., 2024	1
Park et al., 2023	1
Gao & Liu 2021	1
Shimkhada & Ponce, 2022	1
Ho et al., 2023	1
Layug et al., 2022	7
Liu & Modir 2020	1
McGarity-Palmer et al., 2024	1
Chae et al., 2021	1

Corpuz 2021	3
Ratcliff et al., 2023	4
Dhanani & Franz 2021	3
Huang & Liu 2020	2

Articles Excluded by Full-Text (n=17):

Lee et al., 2021	1
Sadri et al., 2024	4
Lantz & Wenger 2023	1
Wang 2024	1
Fan 2022	1
Cao et al., 2023	2
Park et al., 2021	3
Wayne et al., 2021	5
Lee 2020	1
Tsai et al., 2020	2
Hswen 2022	2
McGarity-Palmer et al., 2024	1
Butcher 2020	5
Anon 2021	5
Stewart & Beckman 2023	2
Le 2021	1
Young & Cho 2021	4

Exclusion criteria for articles excluded by abstract: 1 - Does not mention social media; 2 - Does not mention mental health outcomes/effects; 3 - Does not mention mental health outcomes/effects; Not based in U.S.; 4 - Does not mention social media AND does not mention mental health; 5 - Not a peer-reviewed article; 6 - Not based in U.S.; 7 - Not specific to the COVID-19 pandemic; 8 - Lapse in judgement

Exclusion criteria for articles excluded by full-text: 1 - Does not mention social media; 2 - Does not mention mental health outcomes/effects; 3 - Does not mention mental health outcomes/effects; Not based in U.S.; 4 - Does not mention social media AND does not mention mental health; 5 - Not a peer-reviewed article
