# Supplementary material for: Anti-Asian sentiment on social media and mental health among Asian and Asian American populations in the United States during the COVID-19 pandemic: A systematic literature review
Source: Soc Sci Humanit Open. Author manuscript; Available in PMC 2025 Dec 31. (PMC12753001; doi:10.1016/j.ssaho.2025.102282)
Supplement: 3 [file NIHMS2130948-supplement-3.pdf]

Appendix C. Quality assessment for 15 selected articles using AXIS tool

**Quality assessment criteria (AXIS tool)**	1. Clear study objectives	2. Study design appropriate	3. Sample size justified	4. Target population clearly defined	4. Target population clearly defined	6. Selection process appropriate (sample)	7. Measures undertaken to adresss non-response rate	8. Outcome variables appropriate	9. Measurements validated/piloted or published	10. Statistical significance clearly presented	11. Methods sufficiently described	12. Basic data adequately described	13. Response rate does not raise concerns about non-response bias	14. Non-responders described	15. Results internally consistent	16. results from all analyses described	17. Discussions and conclusions justified by results	18. Limitations discussed	19. Absence of conflict of interests	20. Ethical approval and participant consent	Total score (/20)	**Total score (%)**
Ng, R. et al., 2024	yes	yes	yes	yes	yes	yes	not relevant	yes	yes	yes	yes	yes	not relevant	not relevant	yes	yes	yes	yes	yes	not reported	16/20	**80**
Jacques, E. et al., 2023	yes	yes	yes	yes	yes	yes	not relevant	yes	yes	yes	yes	yes	not relevant	not relevant	yes	yes	yes	yes	yes	not reported	16/20	**80**
Nguyen, T. T., et al., 2024	yes	yes	yes	yes	yes	yes	not relevant	not reported	no	yes	yes	yes	not relevant	not reported	yes	yes	yes	yes	yes	not reported	14/20	**70**
Darling-Hammond, S., et al., 2020	yes	yes	no	yes	yes	yes	not relevant	yes	not reported	yes	yes	yes	not reported	not reported	yes	yes	yes	yes	yes	not reported	14/20	**70**
Wheeler, B., et al., 2023	yes	yes	yes	yes	yes	no	not relevant	yes	yes	yes	yes	yes	not relevant	not relevant	yes	no	yes	yes	not reported	not reported	13/20	**65**
Chen W. T., et al., 2022	yes	yes	not relevant	yes	not relevant	not relevant	not relevant	yes	not relevant	not relevant	not relevant	yes	not relevant	not relevant	yes	not relevant	yes	no	not reported	not relevant	7/20	**35**
Hswen, Y., et al., 2021	yes	yes	not reported	yes	yes	not reported	not relevant	yes	yes	yes	yes	yes	not relevant	not relevant	yes	yes	yes	yes	yes	not reported	14/20	**70**
Yang, C. C. et al., 2020	yes	yes	not reported	yes	yes	yes	no	yes	yes	yes	yes	yes	not reported	no	yes	yes	yes	yes	not reported	yes	15/20	**75**
ElTohamy, A., et al., 2024	yes	yes	not reported	yes	yes	yes	yes	yes	yes	yes	yes	yes	not reported	yes	yes	yes	yes	yes	yes	yes	18/20	**90**
Pan, S., et al., 2021	yes	yes	yes	yes	yes	yes	yes	yes	yes	yes	yes	yes	not reported	yes	yes	yes	yes	yes	yes	yes	19/20	**95**
Nguyen, T. T., et al., 2020	yes	yes	yes	yes	yes	not reported	not relevant	yes	yes	yes	yes	yes	not relevant	not reported	yes	yes	yes	yes	yes	not reported	15/20	**75**
Wei, H., et al., 2024	yes	yes	yes	yes	yes	yes	not relevant	yes	yes	yes	yes	yes	not relevant	not reported	yes	yes	yes	yes	yes	yes	17/20	**85**
Huang, X., et al., 2022	yes	yes	not reported	no	not relevant	not reported	not relevant	yes	no	not relevant	yes	yes	not relevant	not relevant	yes	yes	yes	no	yes	not relevant	17/20	**85**
Hohl, A., et al., 2020	yes	yes	yes	yes	yes	no	not relevant	yes	yes	yes	yes	yes	not relevant	not relevant	yes	no	yes	yes	yes	not reported	14/20	**70**
Cho, H., et al., 2020	yes	yes	yes	yes	yes	yes	not relevant	yes	yes	yes	yes	yes	not relevant	no	yes	yes	yes	yes	yes	not reported	16/20	**80**
**Mean score (%)**																						**75**
